# How to deal with mind-reading technologies

**DOI:** 10.3389/fpsyg.2023.1290478

**Published:** 2023-11-14

**Authors:** Roberto Andorno, Andrea Lavazza

**Affiliations:** ^1^Institute of Biomedical Ethics and History of Medicine, University of Zurich, Zürich, Switzerland; ^2^Centro Universitario Internazionale, Arezzo, Italy; ^3^Department of Brain and Behavioral Sciences, University of Pavia, Pavia, Italy

**Keywords:** mental privacy, mind-reading technologies, dual-use technologies, human rights, brain data

## Introduction

In his famous dystopian novel *Nineteen Eighty-Four*, published in 1949, George Orwell depicts a totalitarian society where citizens are under constant surveillance by the authorities (Orwell, 1989). The two protagonists, Winston and Julia, secretly conspire against the state personified by Big Brother. At some point, Julia tells Winston: “They can make you say anything—*anything*—but they can't make you believe it. They can't get inside you” (p. 174). Julia and Winston are talking about what might happen to them once the so-called Thought Police has arrested them. Julia believes, and Winston agrees, that although the Thought Police can torture them in different ways, they will always have that ultimate refuge of their freedom—their minds—as no one can have direct access to their thoughts. Winston concludes that “with all their cleverness they had never mastered the secret of finding out what another human being was thinking” (p. 174). However, Winston and Julia are wrong. It is only after being arrested they realize to their horror that their most inner thoughts have indeed been deciphered by the authorities. During the final scenes of the novel, his tormentor O'Brien tells Winston exactly what he is thinking, sometimes reproducing his internal monolog word for word.

The ability to read thoughts, once beyond reach in Orwell's time, is gradually becoming a reality through brain imaging technologies such as functional magnetic resonance imaging (fMRI). This procedure is primarily used to localize and measure brain activity with the goal of diagnosing neurological disorders. In the clinical setting, fMRI serves a variety of purposes including preoperative risk assessment of brain surgery (Luna et al., 2021), and functional mapping of brain areas to detect functional abnormalities or to monitor patients' post-stroke or post-operative recovery (Crofts et al., 2020). In recent years, there has been growing interest in the use of this technique to decode people's thoughts and intentions in order to enable communication for those who have lost the ability to express themselves verbally due to a variety of neurological conditions. Recent studies under the generic umbrella term of mind-reading include two types of techniques. One is based on the detection of the electrical signals for muscles that gives rise to phonation, including lips, tongue, and jaw (Metzger et al., 2023). The other decodes the brain activity that correlates with the manifestation of thoughts (Tang et al., 2023). Thanks to expert software trained on the individual who is the subject of the experiment, it is also possible to reconstruct verbalizations, images and even the music being heard by the subject (Bellier et al., 2023).

Although the possibility of reading thoughts, memories and intentions is still in its very early stages and current results of such attempts are still inaccurate, the prospect that it may become a reality in the not-too-distant future raises obvious concerns regarding privacy issues. Indeed, the technique could theoretically be used to reveal people's thoughts without their consent, and even for malicious purposes, such as blackmail and discrimination. Certainly, neurotechnologies raise a broad spectrum of ethical and legal issues that go far beyond mental privacy, such as new threats to mental integrity (Lavazza and Giorgi, 2023), freedom of thought and personal identity. However, due to limited space, this opinion article focuses only on mental privacy. It is crucial to stress that mental privacy is a value of fundamental importance to individuals and society. Indeed, our personal freedom is largely dependent on this inner realm of cognition that no one in principle is allowed to invade.

## The dual-use nature of mind-reading technologies

Mind-reading devices are a paradigmatic example of dual-use technologies, as they can be used both to greatly help neurological patients and to seriously harm individuals and society (Andorno, 2022). Two risks in particular can be observed here: first, patients may be induced by their own disabling conditions to accept clinical protocols that lack effective safeguards for the protection of their mental privacy. Think also about predictive neurotechnologies that can detect the onset of epileptic seizures or depressive symptoms by recording deep neural activity, and alert the patient to take necessary precautions. Although these technologies are incredibly useful, they can put patient privacy at risk and may lead to discrimination against people with disabilities (Tacca and Gilbert, 2023). This is why it is crucial to incorporate serious privacy protections in the development of medical technologies and to involve in this effort all stakeholders, including technology designers themselves.

Second, there is a subtle risk that mind-reading techniques could become quickly widely used and successful in various fields before adequate legal measures are implemented. This could lead to a culture where privacy violations are gradually tolerated, similarly as it happened over the past two decades with the rapid success of social media and the tendency of many users to overlook the privacy of their personal data. It is true that current consumer wearable devices in this area are EEG-based and are used to monitor mental states (depression, stress, and level of concentration) rather than, strictly speaking, for “mind-reading” purposes. However, the fact is that technological advances are leading to a decrease in the costs and size of brain imaging tools. As a result, it is highly possible that within the next decade or so, wearable mind-reading devices will become commonplace, much like social media is today. In this context, there exists a genuine risk that users of such devices may not prioritize the confidentiality of their brain data.

It is true that fMRI is not yet advanced enough to be used for widespread and accurate mind-reading, and that it would be difficult to perform it without people's cooperation (Reardon, 2023). However, as the technology continues to develop at a rapid pace, the risks of violation of mental privacy may become a reality soon. For instance, although today the neurological correlations to mental activity that can be identified through fMRI are specific to every individual (“brain fingerprint”), a study has shown that the use of AI tools may help to identify similarities in brain activity patterns of different individuals and lead to the development of a kind of universal mind-reading tool (Chen et al., 2017).

More recently, researchers from the University of Texas at Austin have reported that while fMRI is at present only able to decode a small set of words or phrases, a new AI tool known as “semantic decoder” allows the reconstruction of continuous language, that is, longer sequences of words (Tang et al., 2023). Regarding the argument that mind-reading is not to be feared because it cannot be performed without the individual's cooperation, let us point out that brain data initially collected for clinical or research purposes with the individual's consent could perfectly be misused later for malevolent purposes. It is also possible that such data are collected under some form of coercion, for instance, from people in an employment relationship, where the individuals' cooperation may only appear to be voluntary (Muhl and Andorno, 2023).

## Possible measures to protect mental privacy

What ethical approaches can contribute to safeguarding mental privacy? There are two distinct, complementary models that can be used to achieve this objective. The first model, referred to as “embedded ethics,” involves integrating specific safeguards into the design and production of neurodevices on the initiative of scientists and developers themselves.

The second model can be called “adversarial ethics,” in which external parties, such as lawmakers and civil society, require researchers to comply with certain ethical and legal standards. It is clear that in light of potential threats to mental privacy, the adoption of some legal measures will be necessary in the coming years. In this regard, the formal recognition of a right to mental privacy, as proposed by several authors (Ienca and Andorno, 2017; Yuste et al., 2017; Lavazza, 2018) could contribute to mitigating the misuse of mind-reading technologies.

However, the mere formal recognition of such a right would be largely ineffective without concrete legal measures from civil, criminal, and labor law. To be more precise, legal regulations should require the free and specific informed consent of individuals for the collection and use of their brain data. Simultaneously, it would be beneficial if data protection laws explicitly stated that mental data falls under the category of sensitive personal information. This would ensure that enhanced security measures are put in place to prevent unauthorized third parties from accessing the identity of individuals whose data is being protected.

Of course, there are many particular issues regarding mental privacy that would need to be explored. For instance, we may discuss the acceptability or not of using mind-reading in forensics to prevent lying by defendants and witnesses. Why not also using the technology in the selection of candidates for important public positions? Should not a presidential candidate, who will have the power to impose new taxes or wage a war if elected, be as transparent as possible with their constituencies? These and other similar hypothetical scenarios may sound dystopian today, but they are now within the realm of possibility and need to be seriously considered.

In addition to the measures suggested above, it would be advisable to establish a mechanism for the effective judicial protection of mental privacy. In this regard, on the model of *habeas corpus* and *habeas data*, it is worth considering the proposals made in recent years for the recognition of a so-called “habeas mentem” or “habeas cogitationem” action (from “cogitation”: thought), which would function as a procedural and urgent tool to enforce the guarantees related to the right to mental privacy as well as other rights related to neurotechnologies (Muñoz and Marinaro, forthcoming; Stanzione, 2021).

In recent literature, scenarios have been presented where technology is even more invasive than that available to Big Brother. As a result, it has become urgent to consider ways in which society can deal with mind-reading technologies in a timely and legitimate manner. This is not an attempt to foster prejudice against scientific and technological progress, but rather to safeguard people's right to mental privacy, which is likely to become seriously jeopardized in the coming decades.

## Conclusion

Without delving into the ongoing theoretical debate about whether a right to mental privacy would be a novel right or simply an expansion of the already established right to privacy, this opinion piece aimed to propose some concrete measures that can be taken to reduce the potential threats to mental privacy. However, it is important to consider that criminal groups and undemocratic states may still use mind-reading devices to achieve their nefarious goals. This is why it is imperative that, in parallel to domestic measures, effective international standards and procedures are established to promote respect for people's inner life. Fortunately, various international organizations are already taken the first steps in this direction [UNESCO IBC (International Bioethics Committee), 2021; UN Human Rights Council, 2022].

## Author contributions

RA: Writing—original draft, Writing—review & editing. AL: Writing—review & editing.
